# Public Perceptions of Judges’ Use of AI Tools in Courtroom Decision-Making: An Examination of Legitimacy, Fairness, Trust, and Procedural Justice

**DOI:** 10.3390/bs15040476

**Published:** 2025-04-06

**Authors:** Anna Fine, Emily R. Berthelot, Shawn Marsh

**Affiliations:** Interdisciplinary Social Psychology Ph.D. Program, University of Nevada, Reno, NV 89557, USA; eberthelot@unr.edu (E.R.B.); shawnm@unr.edu (S.M.)

**Keywords:** artificial intelligence, judges, legitimacy, procedural justice, symbolic interactionism

## Abstract

This study examines the role of artificial intelligence (AI) in judicial decision-making, focusing on bail and sentencing contexts. We examined public perceptions of judges who use AI tools compared to those who rely solely on expertise. Using an experimental design, participants (N = 1800; stratified by race/ethnicity and gender) were presented with vignettes depicting judges using varying levels of AI assistance. Key outcomes included perceptions of judicial legitimacy, procedural justice, and trust in AI, with analyses stratified by racial groups (Black, Hispanic, White). The results revealed that judges relying on expertise were generally rated higher in legitimacy than those using AI; however, significant racial differences emerged. Black participants showed greater trust and perceived fairness in AI-augmented decisions compared to White and Hispanic participants. Open-ended responses further highlighted social psychological themes regarding the symbolic meaning of AI in judicial processes. These findings underscore the complexity of integrating AI in the judiciary, emphasizing the need for transparent and equitable implementation strategies to maintain public trust and fairness. Future research should explore underlying factors influencing these perceptions to inform policies that address racial disparities and enhance trust in AI-assisted legal decision-making.

## 1. Introduction

Artificial intelligence (AI) is increasingly becoming an integral part of society, driving advancements and improving efficiency across various sectors. Within the judicial system, AI integration presents both promise and concern. On one hand, AI has the potential to enhance judicial processes, reduce errors, and increase consistency (Aletras et al., 2016). On the other hand, it raises significant concerns about bias, ethics, and the potential reduction in judicial discretion. For instance, recent research has shown that AI models can covertly encode raciolinguistic stereotypes, leading to negative biases against African American English (AAE) speakers that surpass even the most negative stereotypes historically recorded about African Americans (Hofmann et al., 2024). Such biases raise alarms about the fairness of AI when integrated into sensitive areas like judicial decision-making.

As AI tools become more embedded in the judiciary, it is crucial to understand how different groups perceive them and how these tools impact perceptions of procedural justice and legitimacy. This study examines the public’s symbolic perceptions of judges who use AI versus those who rely on their expertise and how these perceptions affect views of procedural justice and legitimacy. Given the historical biases in judicial decisions and AI’s potential to either mitigate or exacerbate these issues, particularly concerning racial and dialectal bias, this research focuses on perceptions among Black, Hispanic, and White individuals.

The decision to include race/ethnicity as an independent variable in this study is rooted in well-documented disparities in how different racial and ethnic groups perceive and experience the judicial system. Historical evidence indicates that Black and Hispanic individuals have consistently reported lower levels of trust in the judicial system compared to White individuals. This distrust is well documented in national surveys and is shaped by both perceived and actual experiences of systemic bias and unequal treatment within the legal system (Peffley & Hurwitz, 2010). For example, Pew survey data show that 84% of Black adults believe they are treated less fairly by the courts and law enforcement compared to White individuals (Pew Research Center, 2019). These perceptions are echoed in findings from the National Center for State Courts (2020), which reported that Black respondents were substantially less comfortable engaging with court processes, including jury service, than White respondents. Notably, such perceptions are grounded in evidence of racial disparities in arrest rates, sentencing outcomes, and incarceration (Peffley & Hurwitz, 2010), contributing to a climate of mistrust toward the justice system.

By including race/ethnicity, we aim to understand whether AI can mitigate these disparities or whether it risks exacerbating existing inequities. The choice to focus on Black, Hispanic, and White participants specifically allows for a targeted comparison: Black and Hispanic individuals represent communities that have been disproportionately affected by biases in judicial decisions, while White individuals generally report more favorable experiences within the justice system. These comparisons help to explore how AI might influence perceptions of fairness, legitimacy, and procedural justice across different racial and ethnic groups, contributing to a more nuanced understanding of AI’s potential role in the judiciary.

AI offers several benefits within the judicial system. It can quickly process vast amounts of information and provide recommendations based on legal concepts and past case outcomes (Panjari, 2023), enhancing judicial decision-making and reducing judges’ cognitive load. More recently, AI in judicial decisions employs algorithms that analyze extensive datasets to assist judges in making informed bail, sentencing, and parole decisions. Proponents argue that AI can reduce errors, streamline processes, and offer objective, data-driven insights to improve decision-making (Zhong et al., 2020). For instance, AI can predict recidivism using historical data, providing judges with more information for their decisions (Bagaric et al., 2019). However, the use of AI in the judiciary is not without significant debate. One primary concern is the potential for AI to inherit and amplify existing biases, potentially leading to discriminatory outcomes and exacerbating the issues it aims to solve (Angwin et al., 2016; Thomson Reuters Institute, 2023). Additionally, the opaque nature of AI raises concerns about transparency and accountability in judicial decisions (Coglianese & Lehr, 2017). Therefore, it is crucial to implement measures to mitigate these biases and ensure that AI is used responsibly in the judicial process (MIT Computational Law Report, 2024).

This paper explores public perceptions of judges who use AI in courtroom decision-making, with particular attention to how these views differ across racial and ethnic groups. Public perception is critical, as it influences how the judicial system is evaluated and trusted. The research is organized into three parts: the first examines bail decisions, the second focuses on sentencing decisions, and the third combines both studies to assess broader patterns. This study addresses the following research questions: (1) How does the public view a judge who uses AI compared to one who relies solely on expertise in bail and sentencing decisions, and how do these views differ among Black, Hispanic, and White individuals? (2) How does the use of AI in these decisions affect perceived legitimacy and procedural justice, and how do these perceptions vary by race/ethnicity? (3) How does a judge’s perceived trust in AI influence public trust in AI, and how does this differ across racial and ethnic groups? (4) What social psychological themes emerge in responses to the open-ended question about judges using AI?

### 1.1. The Role of AI in the Justice System

AI is a tool and a transformative force impacting the legal system in myriad ways. It can revolutionize the criminal justice system by efficiently allocating police resources and identifying potentially dangerous suspects (Simmons, 2018). AI’s application in forensic science is expanding, particularly in areas like image and pattern recognition and 3D crime scene reconstruction (Jadhav et al., 2020). Additionally, AI is being leveraged to predict case outcomes using machine learning (ML) and natural language processing (NLP), thereby enhancing legal reasoning and outcome prediction (Ashley, 2019). NLP can help judges interpret and generate legal documents, summarize case law, and enhance research capabilities (Chowdhary & Chowdhary, 2020; Katz et al., 2023). ML, a branch of AI that improves through data-driven learning, offers the potential for advancing predictive analytics in legal settings, including bail decisions and sentencing (Mahesh, 2020; Simmons, 2018).

AI is playing an increasingly important role in case management and legal research by automating tasks that were traditionally carried out by judges and court staff. This automation can save significant time in handling straightforward cases, allowing judges and staff to focus on more complex matters. As a result, the judicial process becomes more efficient, consistent, and less burdensome for courts and personnel. AI is also being used to improve public access to legal information through chatbots and to support decision-making in legal research, although concerns about bias, transparency, and the need for human oversight remain important (Joint Technology Committee, 2024). In federal courts, AI is being applied to areas such as predictive policing, facial recognition, and medical diagnostics, while also prompting discussions on issues like fairness and the ongoing need for validation (Baker et al., 2023).

The integration of AI into legal research represents a significant shift from traditional methods to more advanced technological approaches, offering greater speed and precision. This transition began in the 1970s with the introduction of digital legal databases such as Westlaw and LexisNexis, marking a major move toward digitalization. The application of Natural Language Processing (NLP) to search complex legal databases (Dixon, 2020; Smith, 2018) further exemplified this change, enhancing the accessibility of legal information and improving the quality of legal practice and education.

AI is evolving from a tool used by judges to an active partner in decision-making, especially in areas like bail and sentencing (Simmons, 2018). The use of AI in judicial decisions, particularly in risk assessments, represents a shift from traditional clinical methods to actuarial approaches. Statistical algorithms now provide more objective and accurate predictions of an individual’s likelihood of reoffending than traditional methods used for bail, sentencing, and parole decisions (Grove et al., 2000; Klingele, 2015; Mayson, 2017; Monahan & Skeem, 2016). This shift toward evidence-based practices seeks to enhance fairness and efficiency in the judicial system by prioritizing data-driven insights over subjective judgment.

### 1.2. Symbolic Interaction Theory

Symbolic interaction theory offers a powerful lens through which to understand how individuals perceive and interpret AI in the context of judicial decision-making. At its core, symbolic interaction theory asserts that meaning is created and modified through social interactions, with symbols and shared understandings playing a pivotal role in shaping individual and group behaviors (Blumer, 1969). This theory emphasizes that people do not react to things or events directly, but rather to the meanings those things have based on social contexts and interactions. In this framework, AI in the courtroom can be understood as a symbol, one that holds different meanings depending on the way it is perceived by various actors involved in the judicial process—judges, lawyers, litigants, and the public.

In the legal context, AI can take on different symbolic meanings, influencing how it is perceived in terms of its fairness, neutrality, and legitimacy. For example, AI might be seen as a symbol of efficiency and impartiality in judicial decision-making. If AI is viewed as a tool that streamlines processes, reduces human error, and makes decisions based on data rather than subjective judgment, it could be associated with increased fairness and transparency. This could lead to greater trust in the judicial system, as the public and legal professionals might feel that AI ensures consistent and rational outcomes in the court system. From this perspective, AI becomes a symbol of progress and trustworthiness, reinforcing perceptions of procedural justice, which refers to the fairness of the processes through which decisions are made (Tyler, 2006b).

On the other hand, the symbolic meaning of AI could also take on a more negative connotation. If AI is associated with biases, lack of transparency, or the dehumanization of judicial processes, it could undermine public trust (Gillespie, 2020). For instance, if people perceive AI as a black-box technology that operates without accountability or as something that reduces human empathy in judicial decisions, it might be seen as a symbol of unfairness or alienation. In this case, AI could evoke feelings of distrust and disconnection, especially among groups who fear that algorithms might perpetuate existing social inequalities. This perception of AI as biased or dehumanizing could be exacerbated by media portrayals of AI as a tool of surveillance or oppression, further impacting public confidence in its use in the courtroom.

Further, fairness is a socially constructed concept determined by an individual’s historical and cultural reality (Berger & Luckmann, 1966), thus public perceptions of fairness in criminal justice research depend on whether AI is seen as working to level the playing field or if it was constructed using data that perpetuate bias. Specifically, a developer’s unconscious bias may factor into design choices that classify Black defendants as higher risk compared to their White counterparts (Dressel & Farid, 2018; Eaglin, 2017). If AI is perceived as replacing human discretion in a dehumanizing way, it may work against the development of trust and legitimacy in the justice system (Kawakami et al., 2022). On the other hand, if AI is perceived as a tool that assists judges in making informed, unbiased decisions, its use may enhance trust and legitimacy (Hannah-Moffat, 2019). The public’s perception of how judges may use AI as a tool in their courtroom decision-making is contingent on how they socially construct the role of AI. These social constructions can be developed based on media narratives (Entman, 1993), including news outlets that may suggest bias exists in the “dirty data” being used to train the AI tools (Richardson, 2019). When the media portrays AI as biased or flawed, the public becomes skeptical (Benjamin, 2019).

Thus, symbolic interaction theory not only helps us understand how AI is perceived in the legal context but also provides insight into how perceptions of fairness and legitimacy are shaped through social interactions and the meanings assigned to technological tools. This perspective is critical for understanding the evolving role of AI in the courtroom, especially as public and professional attitudes toward technology continue to develop.

Public perceptions of AI’s role in the judicial system are critical because they affect the legitimacy and acceptability of judicial decisions. Procedural justice and legitimacy are not merely theoretical concepts but foundational to building public trust in the judicial system. Procedural justice pertains to the perceived fairness of the processes leading to outcomes, while legitimacy refers to the public’s perception of the judicial system’s authority and rightfulness (Tyler, 2006c). Research indicates that when people perceive judicial processes as fair and legitimate, they are more likely to comply with the law and support judicial decisions (Tyler & Huo, 2002). Hence, addressing potential AI-related issues that could harm this trust is essential. This study explores how public perceptions of judges who use AI reflect broader symbolic meanings about authority, fairness, and technology—meanings that may differ across racial and ethnic groups. These symbolic interpretations can also shape how people evaluate the fairness of legal processes, making procedural justice a critical next focus.

### 1.3. Procedural Justice

Procedural justice concerns the fairness of decision-making processes (Tyler, 2006c). It encompasses two main aspects: the process’s fairness and the treatment received from others (Tyler & Blader, 2000). When individuals perceive these aspects as fair, they are more likely to accept social norms and regulate their behavior. People’s perceptions of fairness are influenced by the procedures used to make decisions, even if the outcomes are unfavorable (Thibaut & Walker, 1975).

To uphold procedural justice, it is essential for those affected by a decision to have a voice in the process (Lind & Tyler, 1988). Adherence to principles such as reliable procedures, limited bias, accurate information, the ability to correct wrong decisions, and ethical standards is crucial (Hegtvedt, 2006). These principles can help guide judges in using AI fairly and effectively within the criminal justice system.

AI has the potential to enhance procedural justice by promoting transparency, consistency, and fairness through data-driven methods (Avery et al., 2020). For instance, AI tools can support judges in reducing disparities in sentencing by using standardized algorithms to assess case characteristics, thereby improving consistency across similar cases (Ho et al., 2023). Furthermore, AI systems can enhance transparency by embodying the concept of explainable AI, which ensures that outputs are interpretable (Pillai, 2024), aligning with procedural fairness principles. In practice, AI’s ability to objectively analyze data and assess an individual’s risk level has been integrated into risk assessment tools for pretrial decisions, which aim to minimize human biases related to race or socioeconomic status (Farayola et al., 2023).

Policy recommendations suggest that, for AI to effectively serve these purposes, strong oversight mechanisms are essential. These policies emphasize frequent algorithmic audits, bias mitigation strategies, and transparent reporting requirements to ensure fairness and legitimacy (GAO, 2021). It is important to note that poorly designed or inadequately monitored AI systems can exacerbate existing biases and injustices within the criminal justice system, especially if the data used to train these systems reflect historical disparities (CCJ, 2024). Therefore, proper monitoring and the adoption of ethical guidelines are crucial to ensure AI supports rather than undermines procedural justice (O’Neil, 2016).

Maintaining procedural fairness is vital to ensure public perception of the decision-making process as fair. When the public believes the system operates fairly and justly, they are more likely to accept its decisions and authority (Tyler, 2006c). AI has the potential to enhance procedural justice by increasing consistency and fairness. Implementing ethical guidelines, regulations, and monitoring systems ensures that AI systems are transparent, responsible, and impartial (Acikgoz et al., 2020). Failure to do so could threaten perceived procedural justice and lead to a loss of legitimacy.

### 1.4. Legitimacy

Legitimacy refers to the perceived fairness of authorities, organizations, and social norms (Tyler, 2006a). The degree to which these entities align with a group’s norms, values, and beliefs influences their perceived legitimacy (Zelditch, 2018). Sociological legitimacy involves citizens acknowledging a government’s right to rule (Grossman, 2011). For the judiciary, high levels of legitimacy are essential to uphold the rule of law and maintain political support (Gibson, 2006). Institutional legitimacy reflects the public’s support for the judiciary’s authority, regardless of individual decision outcomes (Audette & Weaver, 2015).

Judicial institutions’ legitimacy depends on their ability to be fair and impartial and interpret the law according to democratic values and citizens’ expectations (Luban, 2008). A lack of institutional legitimacy can undermine the court’s power to enforce its decisions, especially when they contradict public opinion (Bühlmann & Kunz, 2011). In such cases, the public may perceive decisions as influenced by external factors like politics, leading to reduced legitimacy (Badas, 2019). However, this impact is not uniform across all judges. Elected judges, who must campaign and secure votes, are often perceived as more politically influenced than appointed judges, whose legitimacy is tied to their professional qualifications rather than public approval (Bonneau & Hall, 2009). Research suggests that elected judges are more susceptible to perceptions of bias because their decisions may be viewed as catering to constituents or political donors (Gibson, 2012). In contrast, appointed judges, particularly those with lifetime tenure, may be insulated from such concerns, preserving their legitimacy even when making unpopular rulings.

The introduction of AI decision-making tools in the judiciary further complicates legitimacy perceptions. While these tools offer potential benefits, such as efficiency and consistency, they also raise concerns about fairness and bias. Studies have documented that AI systems used in legal decision-making can exhibit racial and gender disparities, often reflecting biases present in the data on which they are trained (Ho et al., 2023). For example, research on risk assessment algorithms used in sentencing and bail determinations has found that these tools can disproportionately classify Black defendants as high-risk while underestimating the risk for White defendants (Angwin et al., 2016). Similarly, gender bias has been observed in AI-driven evaluations, where predictive models may favor male defendants over female defendants due to historical patterns in judicial outcomes (Buolamwini & Gebru, 2018).

Public confidence in AI-assisted judicial decisions may vary depending on whether a judge is elected or appointed. Elected judges, whose legitimacy is already contingent on public opinion, may face greater skepticism when incorporating AI, especially if the technology is perceived as an extension of political or ideological biases. Conversely, appointed judges, particularly those without explicit political affiliations, may be less vulnerable to a “legitimacy penalty” when adopting AI tools. Research suggests that algorithmic decision-making tools are more likely to be perceived as legitimate when they produce favorable outcomes (Martin & Walman, 2022). Thus, for AI to support judicial legitimacy, courts must ensure that these systems are transparent, unbiased, and aligned with principles of procedural fairness. This study investigates the impact of AI application on the perceived legitimacy and procedural justice of bail and sentencing decisions, and whether these effects vary by racial and ethnic group. Understanding these dynamics provides a foundation for exploring a related dimension of public response: trust in AI and in judges who choose to use it.

### 1.5. Trust in AI and Judicial Decision-Making

Trust in AI is a key factor influencing whether individuals view algorithmic tools as legitimate and appropriate for use in courtroom decision-making. In legal contexts, this trust might not only reflect one’s personal beliefs about AI but also be shaped by perceptions of how trusted the technology is by judges themselves. Prior research suggests that trust in technology is not purely performance-based but is socially constructed—shaped by the context of use, the role of the user, and broader perceptions of integrity and purpose (Siau & Wang, 2018; J. D. Lee & See, 2004). In this study, we examine how participants’ trust in AI is shaped by their perceptions of a judge’s trust in AI. This reflects the idea that institutional endorsement or reliance on technology may either reinforce or undermine public trust, depending on pre-existing attitudes and group-based experiences with the justice system.

Importantly, perceptions of AI use and trust may vary across different racial groups, such as in Black and Hispanic communities. As such, a judge’s perceived trust in AI may be interpreted differently across racial groups—potentially signaling legitimacy and innovation for some while raising skepticism or concern for others. By analyzing these group-based differences, this study examines how perceived judicial trust in AI affects participants’ trust in AI among Black, Hispanic, and White respondents.

### 1.6. Racial and Ethnic Disparities in Judicial Perceptions

Racial and ethnic disparities in perceptions of procedural justice and legitimacy are well documented. Minority groups, mainly Black and Hispanic communities, often report lower levels of trust in the judicial system due to historical and ongoing biases (Peffley & Hurwitz, 2010). Research shows that Black individuals have more negative attitudes toward the courts compared to Whites, with Hispanics falling in between (Caldeira & Gibson, 1992; Longazel et al., 2011). These perceptions are crucial to upholding procedural justice and legitimacy principles for all people.

The introduction of AI in judicial decision-making has the potential to either mitigate or exacerbate these disparities, depending on how these tools are perceived and implemented. If AI is used transparently and fairly, it could help rebuild trust among minority groups. However, if AI systems are not adequately monitored and perpetuate bias, they could erode trust in the judiciary. AI has the potential to amplify historical biases within the justice system based on past decisions. Therefore, it is essential for AI to be appropriately monitored, transparent, and used responsibly. AI can contribute to a fairer and more just legal system when implemented correctly.

## 2. Materials and Methods

The purpose of this study was to investigate public perceptions of judges who utilize artificial intelligence (AI) versus those who rely solely on their expertise in making bail and sentencing decisions. Specifically, we examined how these perceptions vary across racial and ethnic groups (Black, Hispanic, and White) and how they relate to perceived legitimacy, procedural justice, symbolic meaning, and trust in AI. Using a between-subjects experimental design, we investigated the following research questions:

1. How does the public symbolically view a judge who uses artificial intelligence compared to one who relies on their expertise in bail and sentencing decisions, and how does this vary among Black, Hispanic, and White individuals?
2. How does the application of AI in bail and sentencing decisions impact perceived legitimacy and procedural justice, and how do these perceptions vary across racial and ethnic groups?
3. How does a judge’s perceived trust in AI influence public trust in AI, and how does this vary among racial and ethnic groups?
4. What social psychological themes emerge in participants’ open-ended responses about judges using AI in decision-making?

### 2.1. Study: Bail (Phase 1) and Sentencing (Phase 2)

#### 2.1.1. Participants

We ran an a priori power analysis conducted using G*Power3 to detect a small effect size of f = 0.15, α = 0.05, with a correlation = 0.05, to have a power of 0.90 to get a total of 864 participants for each phase. Using Prolific.ac—a reliable source for social science research (Peer et al., 2017)—we oversampled for a total of 900 for each phase, to account for participants removed from the sample due to failed manipulation and attention checks (Goodman et al., 2013). We collected a stratified sample of Black, Hispanic, and White individuals with 300 per group. The inclusion criteria required that participants be at least 18 years old and reside in the United States. There were no additional exclusion criteria beyond age and location. Prolific’s prescreening tools were used to ensure participants met these requirements prior to participation.

#### 2.1.2. Phase 1: Bail

For phase 1 (bail) a total of 900 participants completed an online survey via Prolific. The sample consisted of 422 females (47.20%), 447 males (50%), 17 non-binary/third gender (1.90%), and eightwho preferred not to say (0.89%). Participants ranged from 18 to 84 years old (M = 35.98, SD = 13.11). Participants identified as Black (n = 297, 33.11%), Hispanic (n = 301, 33.56%), and White (n = 299, 33.33%). Many participants had a bachelor’s degree (n = 312, 34.78%), followed by some college but no degree (n = 210, 23.41%), high school diploma or GED (n = 137, 15.27%), associate or technical degree (n = 122, 13.60%), master’s degree (n = 81, 9.03%), some high school or less (n = 12, 1.34%), doctoral degree (n = 10, 1.11%), professional degree (JD, MD, DDS) (n = 7, 0.78%), and finally those who preferred not to say (n = 6, 0.67%).

#### 2.1.3. Phase 2: Sentencing

For phase 2 (sentencing), a total of 900 participants completed an online survey via Prolific. The sample consisted of 463 females (51.62%), 410 males (45.71%), 23 non-binary/third gender (2.56%), and onewho preferred not to say (0.11%). Participants ranged from 18 to 78 years old (M = 37.64, SD = 11.96). Participants identified as Black (n = 300, 33.37%), Hispanic (n = 300, 33.37%), and White (n = 299, 33.26%). Many participants had a bachelor’s degree (n = 344, 38.26%), followed by some college but no degree (n = 185, 20.58%), associate or technical degree (n = 119, 13.24%), high school diploma or GED (n = 113, 12.57%), master’s degree (n = 111, 12.35%), doctoral degree (n = 12, 1.33%), some high school or less (n = 8, 0.89%), professional degree (JD, MD, DDS) (n = 6, 0.67%), and finally those who preferred not to say (n = 1, 0.11%).

### 2.2. Design and Procedure

To test our research questions, we conducted an experimental between-subjects design in two phases. The first phase asked about bail and the second phase asked about sentencing. After consenting to participate, participants received a summary of judicial responsibilities. Next, they were randomly assigned to read one of three (bail/sentencing) vignettes: (1) the judge fully relies on their expertise, (2) the judge references an AI tool but also takes into account their expertise, or (3) the judge fully relies on an AI tool (see Appendix A for vignettes). Next, participants filled in a word bank and answered questions about legitimacy, procedural justice, and demographics.

### 2.3. Measures

#### 2.3.1. Symbolic Perceptions

Participants selected words aligned with the vignette they read from a list of words that included positive (e.g., honesty), neutral (e.g., procedure), and negative (e.g., biased) related to procedural justice and legitimacy. The first author coded responses for each word selected: −1 for negative, 0 for neutral, and 1 for positive. The first author calculated a composite score, with higher numbers being positive and lower numbers being negative.

#### 2.3.2. Procedural Justice

Participants were asked a single question, “How fair do you believe the judge’s decision-making process was?” on a five-point Likert scale from 1: Not at all to 5: Completely. Perceived procedural justice of judges was measured using a scale adapted from Tyler and Rasinski (1991), including three statements that reflect perceived procedural justice using a Likert scale ranging from 1: Not at all to 5: Completely (e.g., “Judges seldom consider the views of all sides to an issue before making their decisions”). These four items were combined into a single indicator of procedural justice (α = 0.74).

#### 2.3.3. Legitimacy

First, participants were asked a single question, “How legitimate do you believe the judge’s decision-making process was?” on a five-point Likert scale from 1: Not at all to 5: Completely. Next, the perceived legitimacy of judges was measured using a scale adapted from Cann and Yates (2008) that includes six items that reflect judicial legitimacy using a scale ranging from 1: Not at all to 5: Completely (e.g., “Judges are fair and impartial”). The means and standardized deviations are based on the uncollapsed answers collected on a five-point Likert response set (α = 0.72).

#### 2.3.4. Trust

Participants were asked about their trust in AI and their perceptions of judges’ trust in AI. Specifically, participants were asked, “How much trust do you have in artificial intelligence tools in the justice system at this stage of development?” and “In your opinion, how much trust do you believe judges have in artificial intelligence tools at this stage of development?”

#### 2.3.5. Open-Ended Question

Participants were also presented with an open-ended question asking, “Is there anything else they would like us to know about judges using AI in decision-making?”

### 2.4. Data Analysis and Ethics

All quantitative analyses were conducted using R. For Research Questions 1–3, we used three-way ANOVAs and linear regression models to examine the effects of condition, race/ethnicity, and study phase (bail or sentencing) on perceptions of symbolic meaning, procedural justice, legitimacy, and trust in AI. Post hoc comparisons were conducted using Tukey’s HSD to further explore significant main and interaction effects. Data were cleaned and checked for assumptions of normality and homoscedasticity prior to analysis.

To analyze Research Question 4, we conducted a thematic synthesis of the open-ended responses. First, responses were cleaned to remove empty or irrelevant entries (e.g., “N/A”, “none”), and the remaining data were compiled into a single text corpus. We used ChatGPT-4 (OpenAI, 2023) as an assistive tool to help identify common ideas and illustrative language relevant to this study. Specifically, we prompted the model with instructions, such as the following statement: “Please identify key themes related to how participants view judges who use artificial intelligence in courtroom decision-making. Focus on ideas related to trust, fairness, legitimacy, bias, and symbolism. Provide a list of themes with brief descriptions and representative quotes”. Additional prompts were used to clarify ambiguous content, explore opposing viewpoints, and identify quotes that captured the tone or concerns of different participant perspectives.

The model generated a list of preliminary themes and example quotes, which were then reviewed and refined by the first author. This review involved comparing the model’s output to the raw data to ensure alignment with the research questions and to remove or revise any language that lacked accuracy or nuance. The final synthesis was organized narratively to reflect overarching patterns in participant responses.

It is important to note that this was not a formal qualitative analysis, such as grounded theory or interpretative phenomenological analysis. Rather, it was an exploratory synthesis aimed at capturing broad themes and symbolic meanings present in the data. In addition, ChatGPT-4 was used to assist with editing for clarity and improving the flow of the manuscript. All content and interpretations were critically reviewed and finalized by the author.

## 3. Results

### 3.1. R1. How Does the Public Symbolically View a Judge Who Uses Artificial Intelligence Compared to Their Expertise in Bail and Sentencing Decisions, and How Does This Vary by Black, Hispanic, and White Individuals?

A three-way ANOVA was conducted to examine the relationship between the variables. The dependent variable was symbolic perceptions, and the predictors included condition, race/ethnicity, and study. The interaction effect between condition, race/ethnicity, and study was not significant, *F*(4, 1776) = 0.936, *p* = 0.442. The two-way interaction between race/ethnicity and study was not significant, *F*(2, 1776) = 0.980, *p* = 0.376. The interaction between race/ethnicity and condition was not significant, *F*(4, 1776) = 1.009, *p* = 0.401. The interaction between condition and study was significant, *F*(2, 1776) = 4.885, *p* = 0.01 (see Table 1). To identify which levels were significant, a *t*-test was conducted for study at each level of condition. Individuals had lower views of the judge who used AI in sentencing compared to bail (*p* < 0.01).

The results indicated a significant main effect of condition, *F*(2, 1776) = 234.92, *p* < 0.001. Post hoc comparisons using Tukey’s HSD test indicated that the mean score for the expertise condition (*M* = 7.20, *SD* = 3.17) was significantly higher than the expertise and AI condition (*M* = 5.59, *SD* = 4.78, *p* < 0.001), which was also significantly higher than the AI condition (*M* = 1.41, *SD* = 5.80, *p* < 0.001). The main effect of race/ethnicity was also significant, *F*(2, 1776) = 4.133, *p* < 0.05. Post hoc comparisons using Tukey’s HSD test indicated that the mean score for Hispanic individuals (*M* = 4.35, *SD* = 5.49) was significantly lower than for White individuals (*M* = 5.17; *SD* = 5.22). The main effect of study was not significant, *F*(1, 1776) = 3.72, *p* = 0.053.

### 3.2. R2. How Does the Application of AI in Bail and Sentencing Decisions Impact the Perceived Legitimacy and Procedural Justice, and How Does This Vary by Black, Hispanic, and White Individuals?

A three-way ANOVA was conducted to examine the relationship between the variables. The dependent variable was the Legitimacy Scale, and the predictors included condition, race/ethnicity, and study. The three-way interaction was not significant, *F*(4, 1776) = 1.716, *p* = 0.144. The two-way interactions between condition and race/ethnicity (F(4, 1776) = 1.558, *p* = 0.183), condition and study (*F*(2, 1776) = 0.174, *p* = 0.840), and race/ethnicity and study (*F*(2, 1776) = 0.097, *p* = 0.908) were all not significant.

There was a significant main effect of condition, *F*(2, 1776) = 26.878, *p* < 0.001. Post hoc comparisons using Tukey’s HSD test indicated that the mean legitimacy score for the expertise condition (*M* = 3.32, *SD* = 0.41) was significantly higher than the expertise and AI condition (*M* = 3.24, *SD* = 0.40, *p* < 0.001), which was significantly higher than the AI condition (*M* = 3.14; *SD* = 0.47, *p* < 0.001).

There was also a significant main effect of race/ethnicity, *F*(2, 1776) = 11.310, *p* < 0.001. Post hoc comparisons using Tukey’s HSD test indicated that the mean legitimacy score for the Black group (*M* = 3.30, *SD* = 0.48) was significantly higher than the White (*M* = 3.21, *SD* = 0.40, *p* < 0.001) and Hispanic groups (*M* = 3.19, *SD* = 0.40, *p* < 0.01).

Finally, there was a significant main effect of study, *F*(2, 1776) = 4.341, *p* < 0.05. Post hoc comparisons using Tukey’s HSD test indicated that the mean legitimacy score was higher for the sentencing study (*M* = 3.25, *SD* = 0.44) compared to the bail study (*M* = 3.21, *SD* = 0.44, *p* < 0.05).

#### Procedural Scale

A three-way ANOVA was conducted to examine the relationship between the variables. The dependent variable was the procedural justice scale and the predictors included condition, race/ethnicity, and study. The three-way interaction was significant, *F*(4, 1776) = 4.810, *p* < 0.001. The two-way interaction between race/ethnicity and study was also significant, *F*(2, 1776) = 3.580, *p* < 0.05. To identify which levels were significant, a t-test was conducted for study at each level of study. Black individuals rated procedural justice higher in sentencing compared to bail (*p* = 0.001).

The two-way interaction between race/ethnicity and condition, *F*(4, 1776) = 2.036, *p* = 0.087, and condition and study, *F*(2, 1776) = 1.960, *p* = 0.141, were not significant. There was a significant main effect of condition, *F*(2, 1776) = 30.627, *p* < 0.001. Perceived procedural justice was higher for expertise (*M* = 3.44, *SD* = 0.47) than expertise and AI (*M* = 3.35, *SD* = 0.48, *p* < 0.01), which was higher than AI (*M* = 3.21, *SD* = 0.60, *p* < 0.001).

**Figure 1:**
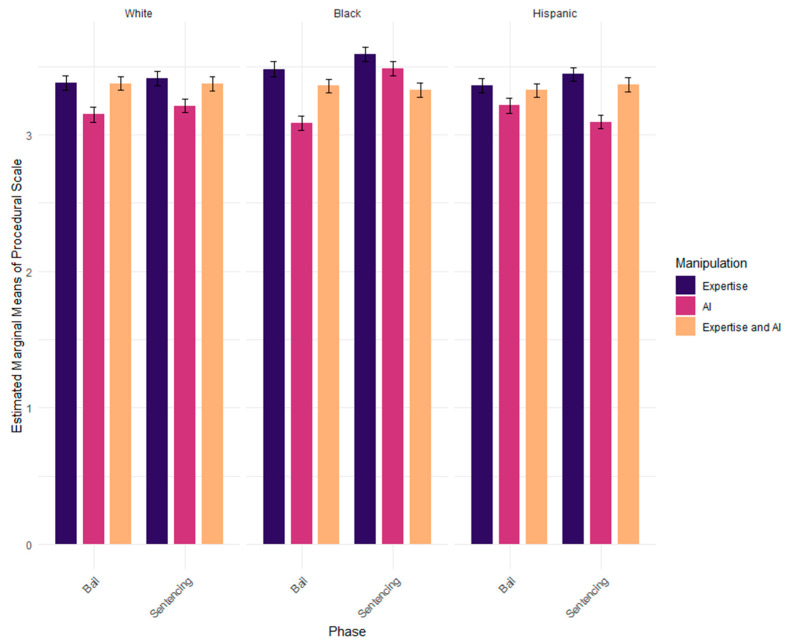
Three-way interaction between study, condition, and race/ethnicity.

There was also a significant effect of race/ethnicity, *F*(4, 1776) = 5.032, *p* < 0.01. Black individuals (*M* = 3.39, *SD* = 0.57) rated the judge with higher procedural justice compared to White (*M* = 3.32, *SD* = 0.45, *p* < 0.05) and Hispanic individuals (*M* = 3.30, *SD* = 0.55, *p* < 0.01). Finally, there was a significant main effect of study, *F*(1, 1776) = 6.728, *p* < 0.01. Procedural justice ratings were higher in sentencing (*M* = 3.37, *SD* = 0.53) than they were for bail (*M* = 3.31, *SD* = 0.52, *p* < 0.01) (Figure 1, Table 2 and Table 3).

### 3.3. R3. How Does Perceived Judges’ Trust in AI Influence Participants’ Trust in AI, and How Does This Vary by Black, Hispanic, and White Individuals?

**Figure 2:**
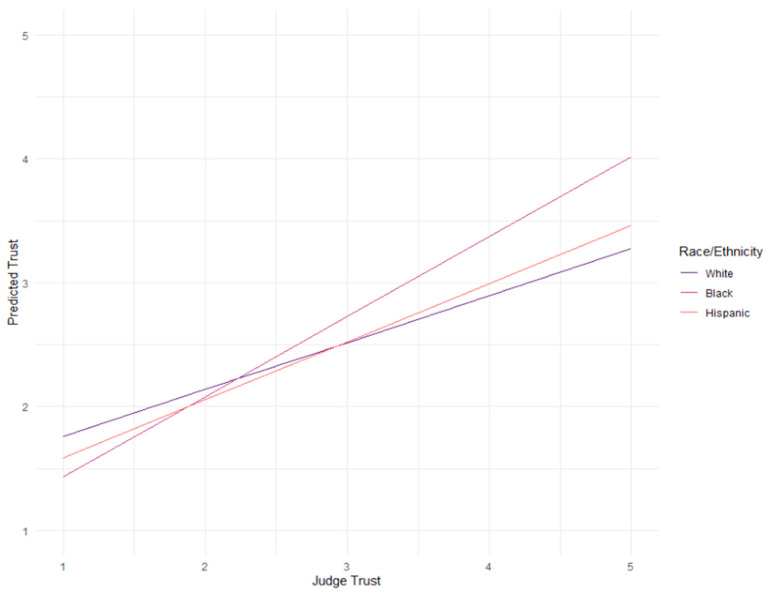
Judges’ trust in AI as a predictor for participants’ trust in AI moderated by race/ethnicity for both bail and sentencing decisions.

The linear regression analysis assessed the effect of judges’ trust on participants’ trust, moderated by ethnicity (Table 4; Figure 2). The overall model was statistically significant, *F*(5, 1788) = 122.1, *p* < 0.001, and accounted for approximately 25.46% of the variance in trust (*R*^2^ = 0.2546, Adjusted *R*^2^ = 0.2525).

The regression analysis revealed a significant main effect of perceived judges’ trust in AI tools (judge trust) on participants’ trust in AI tools (trust), with an estimated coefficient of 0.37827 (*p* < 0.001). This indicates that as participants perceive judges to have higher trust in AI tools, their own trust in these tools increases.

The interaction between judge trust and race/ethnicity revealed that the relationship between judge trust and trust was significantly stronger for Black individuals compared to White individuals, *b* = 0.26663, *t*(1788) = 4.848, *p* < 0.001. These results suggest that race/ethnicity moderates the effect of judge trust on trust, with the relationship being particularly pronounced among Black individuals.

### 3.4. R4. What Themes Are Found Within the Open-Ended Questions?

Open-ended responses revealed a range of views on the role of AI in judicial decision-making. A few participants described AI as potentially helpful for increasing efficiency, particularly in routine processes like bail determinations. One respondent remarked that AI could be useful “for the reason of determining bail”, and another noted that it was acceptable “as long as their own expertise and thoughts are the main reasonings for their decisions”.

Concerns were also expressed about the possibility that AI could replicate or reinforce biases present in its training data or design. One participant observed, “AI can be a great tool, but where it gets its information from will likely inherently carry a bias”. Another commented, “AI can be biased based on what a programmer enters into the system”, raising concerns about unintended bias going undetected.

Several responses reflected skepticism about AI’s capacity to handle complex judicial contexts. One individual wrote, “AI can’t determine any human side of any case. Completely unacceptable”, while another stated, “AI does not allow for decisions based on mitigating or aggravating circumstances”, pointing to perceived limitations in judgment and empathy. AI’s reliance on historical data also raised concerns about the potential to perpetuate existing injustices. One participant noted, “Too many incorrectly ruled cases would be used to help AI make decisions”, and another warned that decisions based on biased criminal histories would “also be biased”.

In addition to concerns, some responses suggested that AI could play a supportive role if used transparently and with appropriate oversight. One person explained, “Judges should use artificial intelligence to help guide their decisions but they shouldn’t base their final decision on the AI’s suggested action”. Others emphasized the importance of safeguards, with one participant writing, “Judges using AI in decision-making need to be extremely careful that they don’t make a mistake and ruin someone’s life by misusing AI”.

Overall, the synthesis reflects a mixture of caution, criticism, and conditional support for AI’s use in judicial contexts. While not a formal qualitative analysis, these responses offer insight into public perceptions of AI’s potential benefits and risks, particularly in relation to fairness, bias, and the role of human judgment.

## 4. Discussion

This combined study aimed to explore how the public views judges who use their expertise compared to those who use AI in both bail and sentencing decisions. The focus was on understanding these perceptions across Black, Hispanic, and White individuals. The findings provide comprehensive insights into symbolic perceptions, legitimacy, procedural justice, and trust in AI, along with an analysis of open-ended responses.

### 4.1. RQ1. Symbolic Perceptions

The results indicate that judges who rely solely on their expertise are perceived more favorably than those using AI, either entirely or in combination with expertise. This pattern persists across bail and sentencing decisions, with AI being viewed more negatively in sentencing contexts. This suggests that the public may associate AI use with reduced human judgment, particularly in high-stakes decisions such as sentencing, reinforcing concerns about AI’s fairness and ethical implications (Coglianese & Lehr, 2017; Simmons, 2018). This finding aligns with previous research suggesting that AI may be seen as beneficial in streamlining less discretionary judicial tasks such as fines and fees (Fine et al., 2024).

### 4.2. RQ2. Perceived Legitimacy and Procedural Justice

Judicial legitimacy, which reflects the perceived fairness and authority of judicial decisions, was significantly higher for judges who relied on their expertise than those who incorporated AI. Notably, Black participants rated judicial legitimacy higher than White and Hispanic participants, suggesting a greater openness to AI-assisted judicial decision-making in communities that have historically experienced biases in the legal system (Peffley & Hurwitz, 2010). These findings align with prior research suggesting that marginalized groups may view AI as a potential mechanism for reducing judicial bias, provided that the AI systems are transparent and equitable (M. K. Lee & Rich, 2021).

Perceptions of procedural justice followed a similar trend, with judges who relied on expertise being rated as fairer than those using AI. However, Black participants rated procedural justice higher than White and Hispanic participants, particularly in sentencing decisions, suggesting they may perceive AI as a tool that could enhance fairness by limiting judicial discretion (Buckland, 2023). These findings highlight the importance of ensuring that AI systems used in judicial decision-making are designed to enhance procedural fairness and mitigate bias (Avery et al., 2020).

### 4.3. RQ3. Trust in AI

Judges’ perceived trust significantly influenced trust in AI. When judges were perceived as trusting AI, participants—especially Black participants—were more likely to trust AI tools in judicial decision-making. This suggests that judicial endorsement plays a critical role in shaping public trust in AI, which has implications for policy implementation and judicial training on AI usage (Fine et al., 2024). Hispanic participants exhibited more cautious attitudes toward AI, reinforcing the need for transparency and public education on AI’s role in legal contexts (Latham & Crockett, 2024).

### 4.4. RQ4. Themes from Open-Ended Responses

The open-ended responses provided deeper insights into public perceptions of AI in judicial decision-making. While some participants acknowledged AI’s potential to enhance efficiency and reduce human error, significant concerns emerged regarding AI’s biases, transparency, and lack of human empathy. Many respondents feared that AI tools could inherit biases from their training data, leading to unfair outcomes (Angwin et al., 2016). Concerns were also raised about AI’s inability to fully grasp mitigating or aggravating circumstances, which are crucial in judicial decision-making (Watamura et al., 2025).

Additionally, participants emphasized the importance of human oversight in AI-assisted decision-making. The prevailing sentiment was that AI should serve as a supplementary tool rather than the primary decision-maker. Calls for transparency and accountability were recurrent themes, reinforcing previous findings that explainable AI and clear audit mechanisms are crucial to public acceptance (Cheong, 2024).

### 4.5. Limitations

This study has several limitations. First, the use of an online convenience sample via Prolific.ac limits the generalizability of the findings to the broader population. Additionally, self-reported measures may be subject to social desirability bias, though the commitment request approach used in data collection helps mitigate this issue (Geisen, 2022). Lastly, while ChatGPT-4 was used to summarize qualitative themes, AI-assisted qualitative analysis may lack the nuance provided by traditional qualitative methods. Future research should employ human-coded qualitative analysis to validate AI-generated themes and explore additional factors influencing public perceptions of AI in judicial decision-making.

Although ChatGPT-4 was used to assist in creating open-ended responses, it does not equate to a formal qualitative analysis. This AI-assisted synthesis may lack the interpretive depth, reflexivity, and transparency typical of traditional qualitative methods. Recent studies indicate that while large language models like ChatGPT can accelerate theme identification and help manage extensive textual datasets, they also present issues related to reproducibility, the risk of content “hallucination”, and a lack of contextualized human interpretation (Perkins & Roe, 2024). In this research, ChatGPT was employed to generate initial summaries, which the researcher subsequently reviewed and refined. However, we recognize that this approach cannot replace the meticulousness of human-coded thematic analysis. Future studies should build on this initial synthesis by employing formal qualitative coding procedures to corroborate and expand upon these results.

Beyond technological limitations, there are conceptual constraints regarding the constructs examined in this study. The operationalization of symbolic perceptions may not fully capture the complexity of how individuals interpret AI’s role in judicial decision-making, as symbolism is highly context-dependent and shaped by social and cultural influences (Blumer, 1969). Furthermore, social interaction theories suggest that trust is built through iterative interactions. Yet the present study relied on a one-time vignette, which may not adequately reflect how trust in AI develops over time (Erfanian et al., 2020).

### 4.6. Implications and Future Directions

The findings of this study have important implications for the integration of AI into the legal system. The general preference for judges to rely on their expertise rather than on AI underscores the need for careful and transparent implementation of AI tools. Ensuring that judges maintain significant control and oversight can help preserve public trust. Additionally, addressing the varying levels of trust and perceptions of fairness across different racial and ethnic groups is essential for promoting equitable justice. Future research should delve deeper into understanding the reasons behind these differences in perceptions and trust. Investigating factors that influence public trust in AI can inform the development of policies and practices that foster acceptance and confidence in the legal system.

## 5. Conclusions

This study explored public perceptions of judges who use their expertise versus those who use AI in making bail and sentencing decisions, focusing on Black, Hispanic, and White individuals. Overall, people tend to view judges who rely solely on their own expertise more positively than those who use AI, either partially or fully. This preference is consistent in both bail and sentencing decisions, with judges relying on their expertise being seen as the most legitimate and fair. Black participants generally rated the legitimacy and procedural justice of judges higher than White and Hispanic participants, regardless of AI involvement, possibly indicating a belief that AI could help reduce biases in judicial decisions.

This study also found that judges’ trust in AI significantly influences participants’ own trust in AI, especially among Black participants. This highlights the importance of judicial endorsement in shaping public trust in AI tools. Open-ended responses revealed concerns about AI’s potential biases and limitations, emphasizing the need for human oversight and transparency in its use.

These findings suggest that while AI can offer benefits in judicial decision-making, its integration must be handled with care and transparency. Ensuring that judges maintain significant control and addressing the varying levels of trust and perceptions of fairness across racial and ethnic groups are essential for promoting equitable justice. Future research should continue to investigate these factors to develop policies that foster public confidence in AI within the legal system.

## Figures and Tables

**Table 1:** Symbolic perceptions of the decision-making process moderated by race/ethnicity for bail and sentencing phase.

Condition	Ethnicity	M	SD
Expertise			
	Bail	6.97	3.42
	Sentencing	7.41	2.90
AI			
	Bail	2.08	5.61
	Sentencing	0.81	5.90
Expertise + AI			
	Bail	5.81	4.46
	Sentencing	5.34	5.12

**Table 2:** Procedural justice ratings for bail.

Condition	Ethnicity	M	SD
Expertise			
	Black	3.48	0.47
	Hispanic	3.36	0.47
	White	3.38	0.38
AI			
	Black	3.09	0.61
	Hispanic	3.21	0.63
	White	3.15	0.55
Expertise + AI			
	Black	3.36	0.50
	Hispanic	3.33	0.56
	White	3.38	0.40

**Table 3:** Procedural justice ratings for sentencing.

Condition	Ethnicity	M	SD
Expertise			
	Black	3.59	0.55
	Hispanic	3.44	0.53
	White	3.41	0.33
AI			
	Black	3.49	0.57
	Hispanic	3.09	0.59
	White	3.21	0.56
Expertise + AI			
	Black	3.32	0.56
	Hispanic	3.37	0.47
	White	3.37	0.37

**Table 4:** Judges’ trust in AI as a predictor for participants’ trust in AI moderated by race/ethnicity for both bail and sentencing decisions.

Condition	Estimate	Std. Error	*t*-Value	*p*-Value
(Intercept)	1.38	0.11	12.414	<0.001
Judge trust in AI	0.38	0.04	8.787	<0.001
Black	−0.59	0.15	−4.024	<0.001
Hispanic	−0.26	0.15	−1.757	0.079
Judge trust: Black	0.27	0.06	4.848	<0.001
Judge trust: Hispanic	0.09	0.06	1.567	0.117

## Data Availability

The original data presented in this study are openly available in OSF at https://osf.io/fd8qn/?view_only=562a281186a94149a84ed5ec5d64dc61or, (accessed on 28 February 2025).

## Appendix A

### Appendix A.1. Vignettes for Phase 1 (Bail)

Expertise: Judge Brown is an experienced judge who has presided over numerous criminal cases involving different types of offenses. This judge has developed a keen sense of judgment over the years, having relied on courtroom experience to determine the appropriate bail amount for each defendant. Judge Brown takes into account various factors such as the severity of the crime, the defendant’s criminal history, and the defendant’s ties to the community. Judge Brown also listens carefully to arguments from both the prosecution and defense before making a decision.

To clarify, Judge Brown used only professional expertise to make the bail decision.

AI: Judge Brown is an experienced judge who has presided over numerous criminal cases involving different types of offenses. This judge has been given access to a state-of-the-art artificial intelligence (AI) system to determine bail amounts. Judge Brown inputs information such as the severity of the crime, the defendant’s criminal history, and the defendant’s ties to the community into the system, which then generates a recommended bail amount based on its algorithms. Judge Brown relies solely on this AI system to determine bail and does not use professional judgment or expertise.

To clarify, Judge Brown used only artificial intelligence (AI) to make the bail decision.

Expertise and AI: Judge Brown is an experienced judge who has presided over numerous criminal cases. This judge has been given access to a state-of-the-art artificial intelligence (AI) system to help determine bail amounts. Judge Brown inputs the relevant information into the AI system and considers its recommendations alongside professional judgment and expertise. Judge Brown takes into account the factors suggested by the AI system but ultimately makes the final decision based on a professional analysis of the case. Judge Brown believes that the AI system is a valuable tool that can help in making more informed decisions but does not solely rely on it to make a decision.

To clarify, Judge Brown used artificial intelligence (AI) as a supplemental tool and incorporated professional expertise to make the bail decision.

### Appendix A.2. Vignettes for Phase 2 (Sentencing)

Expertise: Judge Brown is an experienced judge who has presided over numerous criminal cases involving different types of offenses. This judge has developed a keen sense of judgment over the years, having relied on courtroom experience to determine the appropriate sentence for each defendant. Judge Brown takes into account various factors such as the severity of the crime, the defendant’s criminal history, and the defendant’s ties to the community. Judge Brown also listens carefully to arguments from both the prosecution and defense before making a decision.

To clarify, Judge Brown used only professional expertise to make the sentencing decision.

AI: Judge Brown is an experienced judge who has presided over numerous criminal cases involving different types of offenses. This judge has been given access to a state-of-the-art artificial intelligence (AI) system to determine sentence length. Judge Brown inputs information such as the severity of the crime, the defendant’s criminal history, and the defendant’s ties to the community into the system, which then generates a recommended sentence length based on its algorithms. Judge Brown relies solely on this AI system to determine sentence length and does not use professional judgment or expertise.

To clarify, Judge Brown used only artificial intelligence (AI) to make the sentencing decision.

Expertise and AI: Judge Brown is an experienced judge who has presided over numerous criminal cases. This judge has been given access to a state-of-the-art artificial intelligence (AI) system to help determine sentence length. Judge Brown inputs the relevant information into the AI system and considers its recommendations alongside professional judgment and expertise. Judge Brown takes into account the factors suggested by the AI system but ultimately makes the final decision based on a professional analysis of the case. Judge Brown believes that the AI system is a valuable tool that can help in making more informed decisions but does not solely rely on it to make a decision.

To clarify, Judge Brown used artificial intelligence (AI) as a supplemental tool and incorporated professional expertise to make the sentencing decision.
